# Treatment of experimental colitis in mice with LMP-420, an inhibitor of TNF transcription

**DOI:** 10.1186/1476-9255-5-4

**Published:** 2008-03-10

**Authors:** Laura P Hale, George Cianciolo

**Affiliations:** 1 Department of Pathology, Duke University Medical Center, Durham, NC, USA

## Abstract

**Background:**

LMP-420 is a boronic acid-containing purine nucleoside analogue that transcriptionally inhibits TNF production but is non-cytotoxic to TNF-producing cells.

**Methods:**

This study investigated the efficacy of LMP-420 as an anti-inflammatory agent in acute and chronic colitis induced by oral administration of dextran sulfate sodium (DSS) to mice and in chronic colitis following piroxicam administration to IL-10-deficient mice. The severity of colon inflammation was assessed histologically. TNF levels were measured by enzyme immunoassay.

**Results:**

Administration of DSS for 7 days resulted in severe acute colitis that was associated with a marked increase in stool and colon tissue TNF levels. Initiation of therapy with intraperitoneal (i.p.) LMP-420 on day 4 of DSS exposure decreased colonic TNF to near normal levels on day 7. However, neither i.p. nor oral treatment with LMP-420 affected the development or severity of acute DSS colitis. Initiation of LMP-420 therapy after 3 cycles of DSS administration to establish chronic colitis also had no effect on the severity of chronic colitis. Analysis of colonic TNF combined with longitudinal analysis of TNF and TNF receptor (TNF-RII) levels in stool during the development of chronic DSS colitis demonstrated that the initially elevated colonic TNF levels returned to normal despite intense on-going inflammation in mice with chronic colitis. RAG-2^-/- ^mice deficient in T and B cells also developed severe ongoing colitis in response to 3 cycles of DSS, but showed marked differences vs. wild type mice in stool TNF and TNF-RII in response to DSS exposure. Systemic and oral LMP-420 treatment for 16 days decreased colonic TNF levels in IL-10-deficient mice with chronic colitis, with a trend to decreased histologic inflammation for oral LMP-420.

**Conclusion:**

These studies demonstrate that short-term treatment with a transcriptional inhibitor of TNF production can decrease systemic and local colonic levels of TNF but may not decrease the histologic severity of colitis. Longer term studies using colitis models that are more dependent on TNF elevation should be performed to more accurately assess the potential of LMP-420 for therapy of inflammatory bowel disease.

## Introduction

Inflammatory bowel diseases such as Crohn's disease (CD) and ulcerative colitis (UC) are hypothesized to result from abnormal immune responses to antigens derived from intestinal microbiota (reviewed in [1,2]) that are perpetuated by ongoing exposure to these antigens in the intestine. A number of pro-inflammatory cytokines and chemokines have been demonstrated to be elevated in colonic mucosa and/or leukocytes derived from human inflammatory bowel disease (IBD) patients (reviewed in [3,4]). These include IL-1, IL-6, IL-12, IFN-γ, monocyte chemoattractant protein-1 (MCP-1; also called JE or C-C chemokine ligand 2 (CCL2), and tumor necrosis factor (TNF).

TNF is a major regulator of inflammation. The murine/human chimeric monoclonal antibody infliximab (Remicade™; Centocor; Malvern, PA, USA) neutralizes TNF activity by binding with high affinity to both soluble and membrane-bound TNF [5,6]. Infliximab binding to membrane-bound TNF renders those cells susceptible to lysis by complement or effector cells [6]. Infliximab binding also induces apoptosis of activated T cells from CD patients [7]. Etanercept (Enbrel™; Immunex Corp; Seattle, WA, USA) is a dimeric fusion protein consisting of the extracellular ligand binding domain of the human p75 TNF receptor linked to the Fc portion of human IgG1. Etanercept binds specifically to TNF and blocks its interaction with naturally occurring cell surface TNF receptors. Cells expressing transmembrane TNF bind etancercept but are not lysed *in vitro *in the presence or absence of complement [8]. Both the anti-TNF drugs infliximab and etanercept have been shown to be beneficial in rheumatoid arthritis and psoriasis [9-11]. Infliximab has been shown to significantly decrease inflammatory activity and to be an effective maintenance therapy in patients with CD or UC and to enhance closing of fistulas in CD [12-16]. However, an authoritative randomized controlled trial of etanercept failed to demonstrate efficacy in CD when used at the same doses effective for treatment of rheumatoid arthritis [17]. The mechanisms responsible for the differential efficacy of infliximab vs. etanercept in IBD remain unclear. However, destruction of TNF-producing cells by infliximab (but not etanercept) could produce a generalized immunosuppressive effect that might contribute to its efficacy in IBD [7,18].

Disadvantages of infliximab treatment include the high cost of therapy, the need for administration by intravenous infusion, development of anti-chimeric antibodies that limit drug effectiveness, increased susceptibility to severe opportunistic infections [19,20], and the reactivation of tuberculosis [21-23]. Its relatively long plasma half-life (10.5 days) makes it difficult to stop drug action if adverse effects occur. An orally active small molecule that inhibits production of TNF and other pro-inflammatory cytokines without generalized immunosuppression would be predicted to overcome many of the disadvantages associated with currently available TNF antagonists. Because such a drug would also allow determination of how local vs. systemic TNF inhibition and cytotoxicity toward TNF-producing cells contribute to efficacy in IBD therapy, the data generated would also have broad applicability toward improving IBD therapies.

**Figure 1:**
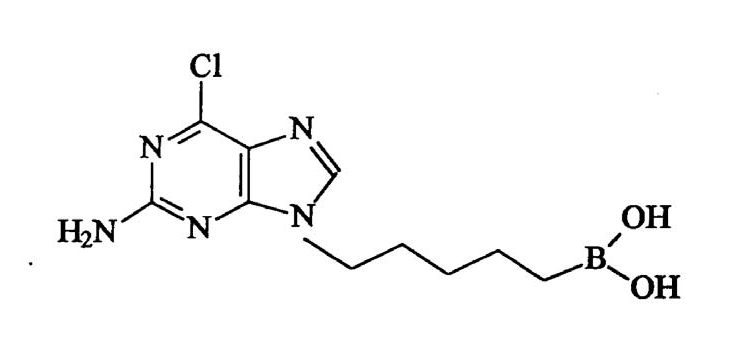
**Chemical structure of LMP-420**. (2-amino-6-chloro-9 [5(dihydroxyborylpentyl]-purine).

LMP-420 (MW = 284.5; Figure 1) is a purine-based small molecule that bears a boronic acid side chain. It is a more potent analogue of a parent compound that was originally identified by a cell-based screen for agents that altered cytokine production by human monocytes [24]. LMP-420 strongly inhibits production of both TNF mRNA and protein [25] and is non-toxic *in vitro *toward cells present in the colon, including lymphocytes, monocytes, and intestinal epithelial cells. It does not bind to or interfere with the activity of pre-formed TNF (unpublished data). LMP-420 can be readily administered by injection. However, its physicochemical stability, combined with a chemical structure favoring retention in the gastrointestinal tract, suggested that LMP-420 might also have direct local activity within the intestine when given orally. Based on its *in vitro *activity profile, we hypothesized that *in vivo *therapy with LMP-420 would decrease intestinal production of pro-inflammatory cytokines including TNF and thus decrease intestinal inflammation in IBD. Here, we present results obtained with the dextran sulfate sodium (DSS) murine models of acute and chronic colitis and chronic colitis in IL-10-deficient (IL-10^-/-^) mice.

## Materials and methods

### Animal studies

Specific pathogen-free wild type female C57BL/6 mice were obtained from Jackson Laboratories (Bar Harbor, ME, USA) and typically used at an age of 6–8 weeks. Starting weights (mean ± SD) for acute and chronic DSS studies were 18.8 ± 1.0 g and 19.2 ± 0.9 g. RAG-2^-/- ^female mice on the C57BL/6 background were obtained from Taconic Farms (Germantown, NY, USA) (mean weight 22.5 ± 1.2 g). Mice were housed in polycarbonate micro-isolator or individually ventilated cages and were allowed access to food and water *ad libitum*. All animal studies were approved by the Duke University Institutional Animal Care and Use Committee.

A bacterial lipopolysaccharide (LPS) challenge model was used to test the ability of LMP-420 to block TNF production *in vivo*. Mice were treated for 16 days with 2 mg (100 mg/kg) LMP-420 given intraperitoneally (i.p.) once daily or with LMP-420 doses of up to 145/mg/kg/day given orally mixed in food. The i.p. dose of LMP-420 used was chosen to represent the highest parenteral dose reasonable given the solubility of the drug (~10 mg/ml in 5% sorbitol). The 16 day treatment period was chosen to assess the toxicity of repeated daily dosing of LMP-420 and because a treatment period of 16 days or longer is typically needed to result in histologically detectable differences in inflammatory activity. Vehicle- or LMP-420 treated mice were challenged with a lethal dose of 0.5 mg LPS (from *E. coli *strain O111:B4; catalog#L-2630, Sigma-Aldrich, St. Louis, MO, USA) given 4 hrs after the last LMP-420 dose. Mice were euthanized 2 hrs after LPS challenge to measure TNF levels in serum and colon tissues.

Acute colitis was induced by addition of 3% DSS (40 kDa molecular weight; obtained from ICN, Costa Mesa, CA, USA) into the drinking water for 7 days. Chronic colitis was induced by 3 cycles of DSS administration, each consisting of 5 days DSS followed by 16 days of recovery. The clinical severity of colitis was assessed by daily observations for weight loss, stool consistency, and the presence of gross bleeding. Freshly passed stool pellets were tested periodically for occult blood using Hemoccult Sensa II cards (Beckman Coulter, Palo Alto, CA, USA).

Specific pathogen and *Helicobacter*-free female IL-10-deficient (IL-10^-/-^) mice on the C57BL/6 background (strain name = B6.129P2-Il10^tm1Cgn^/J; stock # 002251) were obtained from Jackson Laboratories (Bar Harbor, ME, USA). Powdered food containing 200 ppm piroxicam was administered for 7 days at 6 – 7 wks of age (weights = 18.6 ± 1.6 g, mean ± SD) to accelerate the development of chronic colitis [26]. Piroxicam was discontinued and mice were treated for 16 days with a dose range of LMP-420 (0, 5, 15, or 45 mg/kg/day) given i.p. once daily or food containing LMP-420 that delivered mean LMP-420 doses of 0, 41, 62, or 138 mg/kg/day.

### *In vitro *studies

Thioglycollate-stimulated macrophages were obtained for *in vitro *studies by peritoneal lavage (ice-cold PBS; 0.1% BSA; 10 u/ml sodium heparin) of euthanized mice injected intraperitoneally 3–4 days earlier with 1 ml of sterile thioglycollate broth (DIFCO; Voight Global Distribution, Kansas City, MO, USA). Peritoneal exudates were centrifuged, washed once with PBS and resuspended in complete RPMI medium containing 10% heat-inactivated (56°C, 45 min) fetal bovine serum (FBS). Macrophages were isolated by subsequent overnight adherence to plastic by incubation at 37°C in humidified 5% CO_2_, then plated into 96 well plates at 1 × 10^5 ^cells/well in RPMI1640 with 5% heat-inactivated FBS, 100 u/ml penicillin, 100 μg/ml streptomycin and the indicated concentration of LMP-420. Cells were incubated for 2 hrs, LPS (1 μg/ml; from *E. coli *strain O111:B4, Sigma-Aldrich, St. Louis, MO, USA) was added, and after an additional 24 hrs TNF content of the media was measured.

Murine splenocytes were prepared from spleens harvested from normal mice after euthanasia. Briefly, the spleens were removed aseptically and placed in plastic Petri dishes containing ~10 ml of tissue culture medium. The medium was drawn up in a 27 gauge needle on a 10-ml syringe and then repeatedly injected under the splenic capsule, forcing leukocytes from the splenic tissue into the medium. The leukocytes were pelleted by centrifugation (15 min, 200 × g, 4°C), washed once with PBS and contaminating red blood cells removed by a brief (< 20 s) hypotonic lysis in 9 volumes of sterile H_2_O followed by the addition of 1 volume of 10× PBS. The leukocytes were washed once with complete RPMI medium and resuspended to a concentration of 1 × 10^6^/ml. For studies of LPS stimulation, splenocytes were cultured for 2 hrs in 96 well plates at 2 × 10^5 ^cells/well in RPMI1640 with 5% heat-inactivated FBS, 100 u/ml penicillin, 100 μg/ml streptomycin with the indicated concentrations of LMP-420. One μg/ml LPS was added and TNF content of the media was measured after an additional 24 hrs of culture. For studies using CD3 stimulation, splenocytes (1 × 10^6^/ml) were incubated with media (RPMI1640 with 5% heat-inactivated FBS, 100 u/ml penicillin, 100 μg/ml streptomycin) or the indicated concentration of LMP-420 in media for 2 hrs at 37°C in polypropylene tubes and then 200 ml of cell suspension was added to each well of a 96-well BioCoat™ antimurine CD3 plate (BD Biosciences, Franklin Lakes, NJ, USA). The TNF content of culture supernatants was measured at both 24 and 48 hr time points for CD3-stimulated splenocytes.

### Treatment with LMP-420

LMP-420 was custom synthesized by Scynexis Inc. (Research Triangle Park, NC, USA) under provisions of a Material Transfer Agreement between LeukoMed (Raleigh, NC, USA) and Duke University. For i.p. injections, a 10 mg/ml stock solution was prepared in 5% sorbitol, pH 9.0 in sterile water and further diluted as necessary to deliver the desired dose in a volume of 0.2 ml. For oral delivery, LMP-420 was mixed with powdered rodent diet containing 20 μg omeprazole per 3.5 g food to minimize gastric degradation of LMP-420. All control mice in oral dosing studies also received food containing omeprazole. The method of serial dilutions was used to ensure uniform mixing of drugs into powdered food. The weight of food consumed daily was recorded and averaged to determine the mean dose received per kg body weight during therapy. For studies involving both i.p. and oral administration of LMP-420, the doses listed are as administered. Serum and tissue levels of LMP-420 were not measured in this study.

### Tissue analysis

Mice were euthanized by CO_2 _asphyxiation in accordance with the American Veterinary Medical Association Panel on Euthanasia. The entire colon (cecum to anus) was removed and colon length was measured from the ileocecal junction to the anus. The colon was then divided into segments representing the cecum, proximal, mid-, distal, and terminal colon/rectum. Colon tissue obtained from the proximal ends of the mid-, distal, and terminal colon segments was harvested for TNF measurement. Five colors of permanent tissue marking dye (Bradley Products, Bloomington, MN, USA) were used to specifically identify each colonic segment. These tissues were fixed in Carnoy's solution for 2 – 4 hrs, then processed and embedded into paraffin.

The severity of inflammation seen in hematoxylin and eosin-stained sections was scored independently by a pathologist blinded to treatment group. Histologic scores were calculated as described, using a scale that takes into account mucosal changes in 5 different bowel segments, including hyperplasia and ulceration, degree of inflammation, and % of each bowel segment affected by these changes [[26]; modified from [27]]. Using this scale, the maximum score is 75 and a score > 12 indicates colitis.

### Cytokine/cytokine receptor measurements

Stool for cytokine analysis was collected before DSS exposure began (day 0), at the end of each 5 day cycle of DSS administration (day 5, 26, and 47, and after 16 days of recovery prior to beginning the next DSS cycle (days 21, 42, and 63). Freshly obtained stool was kept on ice until it was homogenized at 100 mg stool/ml buffer in PBS containing 1% bovine serum albumin, 0.1% Kathon (a microbiocide; Supelco, Bellefonte, PA, USA), and Protease Inhibitor Cocktail (Sigma-Aldrich, St. Louis, MO, USA). Insoluble material was removed by centrifugation at 4°C at 15,000 × g in a microfuge for 10 min. Stool extracts were filtered at 0.2 μm then stored at -20°C until assayed. Fresh colon samples were homogenized at 100 mg tissue/ml buffer using a PowerGen 125 tissue grinder (Fisher Scientific, Suwanee, GA, USA) and the BioPlex Cell Lysis Kit (BioRad, Hercules, CA, USA) according to the manufacturer's instructions. Colon tissue extracts were then frozen at -20°C until analysis. TNF and TNF-RII were quantitated in tissue culture supernatants, stool, and colon tissue extracts by enzyme immunoassay using Duo-Set reagents (R&D Systems, Minneapolis, MN, USA). Results were expressed as pg/ml for culture supernatants or as pg/100 mg tissue or stool.

### Statistical analysis

Statistical comparison of groups was performed using Student's t test or analysis of variance (ANOVA). A value of p ≤ 0.05 was considered to be significant.

## Results

### LMP-420 inhibits TNF response to LPS *in vitro*

**Figure 2:**
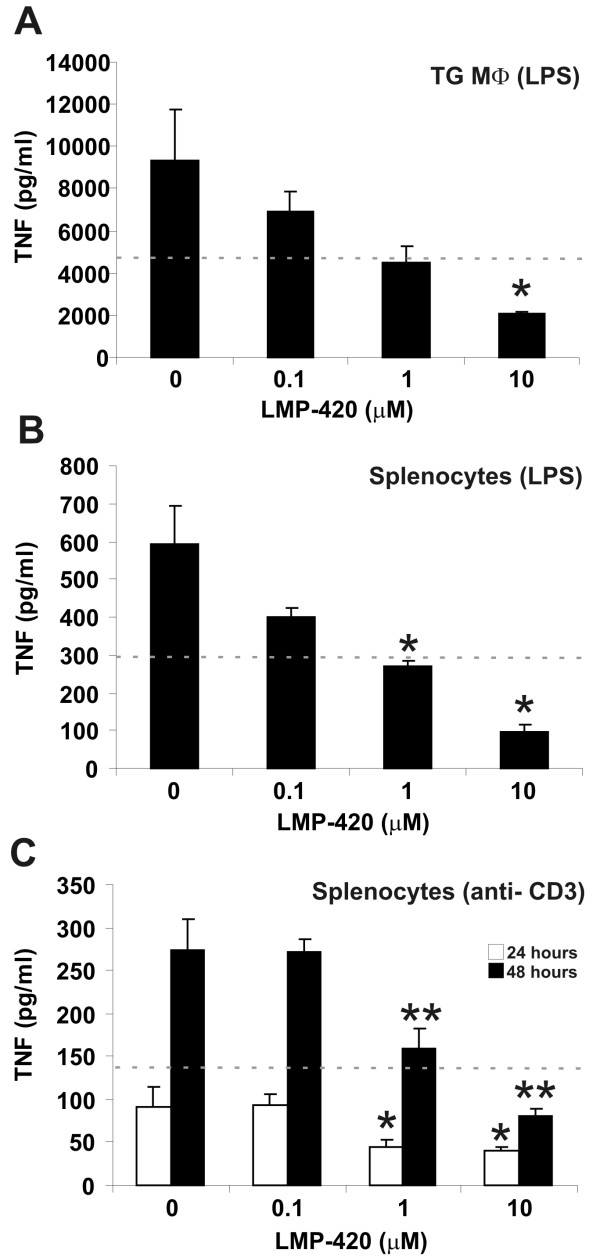
**LMP-420 inhibits production of TNF protein by murine lymphocytes and macrophages**. **A**. A 2 hr pre-treatment with LMP-420 markedly decreases TNF production by thioglycollate-elicited macrophages (MΦ) (n = 3) 24 hrs after exposure to LPS. The IC50 threshold (the concentration that inhibits 50% of the TNF produced) is indicated by the dotted line and is slightly less than 1 μM for this assay. * indicates p ≤ 0.03. **B. **Pre-treatment with LMP-420 also markedly decreases TNF production by splenocytes (n = 3) 24 hrs after LPS exposure. The IC50 threshold for this assay (dotted line) was slightly less than 1 μM. **C**. Pre-treatment with LMP-420 also significantly decreased TNF production by splenocytes (n = 8) at both 24 and 48 hrs after stimulation with CD3 antibody. The IC50 for CD3-stimulated T cells at 48 hrs (dotted line) was slightly greater than 1 μM. * indicates p < 0.03 and ** indicates p ≤ 0.003 vs. CD3-stimulated control not exposed to LMP-420.

LMP-420 very effectively inhibits production of TNF mRNA and protein when applied topically *in vitro *to macrophages and lymphocytes, cell types that are present in the colonic lamina propria (Figure 2). This suggests that if it is not degraded, LMP-420 could have local (topical) activity in the gastrointestinal tract when administered orally. The concentration of LMP-420 required to inhibit 50% of the TNF synthesized (IC_50_) by LPS-stimulated murine monocytes *in vitro *is ~500 nM (Figure 2). The corresponding IC_50 _for TNF production by human peripheral blood mononuclear cells is ~50 nM (25). The molecular basis for the ~10-fold increased sensitivity of human vs. murine cells to LMP-420 is unclear, but suggests that LMP-420 is likely to have greater anti-inflammatory activity in humans than in mice.

### LMP-420 inhibits TNF response to LPS *in vivo*

**Figure 3:**
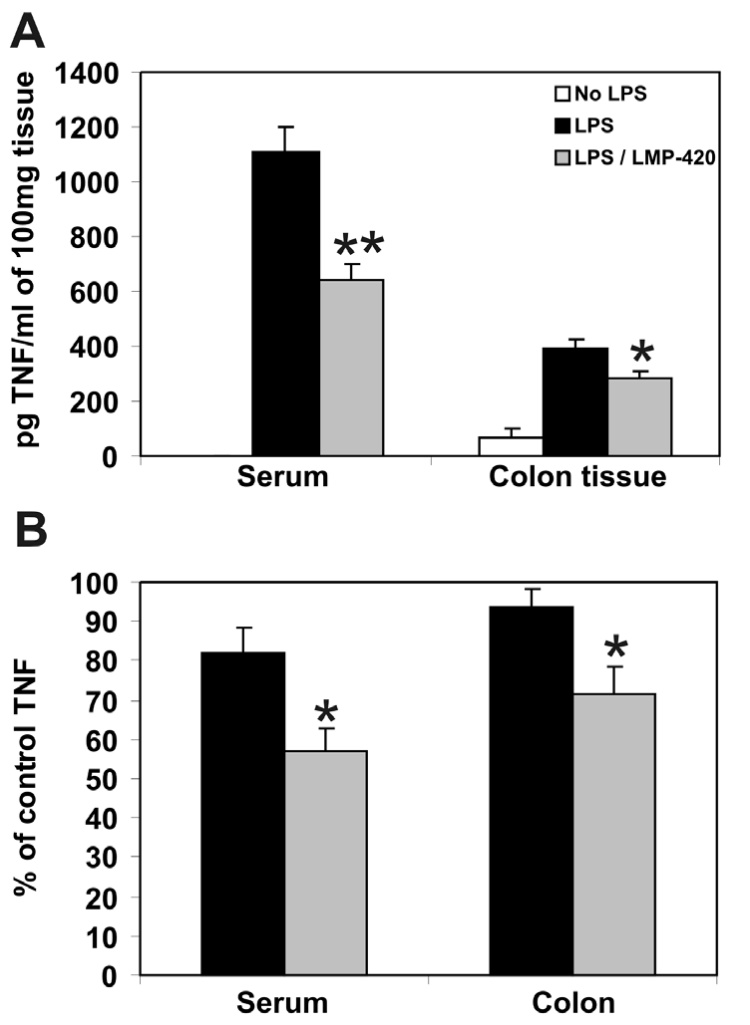
**LMP-420 decreases serum and colonic TNF induced by LPS stimulation *in vivo***. **A**. Mice were pre-treated with 2 mg LMP-420 i.p. for 16 days. Four hours after the last injection, mice were challenged i.p. with a lethal dose of 0.5 mg LPS then euthanized 2 hrs. later for measurement of TNF levels in serum and in colon tissue lysates prepared at 100 mg tissue/ml. Serum TNF was below the limit of detection (< 10 pg/ml) in control mice not exposed to LPS challenge. *p < 0.05 and **p < 0.001 relative to control LPS-stimulated mice. **B**. In 2 separate experiments, groups of 5 mice were pretreated with 75 mg/kg LMP-420 given orally in food for 5 days prior to LPS challenge and TNF measurement. Data is presented as % of control value rather than as absolute numbers because mean serum TNF levels after LPS stimulation of control mice differed markedly (706 vs. 1870 pg/ml) in the 2 experiments. * indicates p < 0.05 compared with control.

Based upon its *in vitro *profile, LMP-420 was hypothesized to be an efficient inhibitor of TNF *in vivo*. The degree of systemic and colonic TNF blockade that resulted from different LMP-420 *in vivo *dosing protocols was first assessed in wild type mice using a bacterial lipopolysaccharide (LPS) challenge model. Mice treated with 2 mg (100 mg/kg) LMP-420 given intraperitoneally (i.p.) experienced transient behavioral depression consistent with hypotension, but recovered to normal behavior within 20 minutes after injection. TNF was below the limit of detection (< 10 pg/ml) in the serum of mice not challenged with LPS. As expected, serum TNF levels were markedly increased to 1109 ± 87 pg/ml (mean ± SEM; n = 9) upon LPS challenge. However, pre-treatment with i.p. LMP-420 decreased LPS-induced serum TNF levels by 42% (Figure 3A; p = 0.0.001) to 641 ± 57 pg/ml serum (n = 3). In contrast to the lack of detectable TNF in the serum of control mice, detectable levels of TNF (66 ± 30 pg/100 mg tissue) were present in colonic tissue of control mice at baseline prior to LPS stimulation. Systemic challenge with LPS increased levels of TNF present in colonic tissue to 388 ± 37 pg/100 mg tissue (n = 9; increase of 488%; p = 0.0.001). Pre-treatment with LMP-420 significantly decreased total colonic TNF content by 28% to 281 ± 27 pg/100 mg tissue (n = 3) (Figure 3A; p = 0.0.045). Although these mice were treated with 2 mg (100 mg/kg) LMP-420 for 16 days prior to challenge, similar levels of TNF inhibition were observed when LPS challenge occurred 4 hrs after a single dose of LMP-420 (data not shown).

Contrary to the toxicity seen when 100 mg/kg LMP-420 was administered i.p., no adverse effects were seen when doses of LMP-420 up to 145 mg/kg/day were administered orally in food for 16 days. LPS challenge was performed for mice that received 75 mg/kg LMP-420 orally in food for 5 days prior to LPS challenge. This oral dose provided similar decreases in LPS-induced serum (-43%) and total colonic TNF levels (-29%; Figure 3B) as were seen with mice given 100 mg/kg via the i.p. route (Figure 3A).

### LMP-420 markedly decreases colonic TNF when given i.p. after initiation of acute DSS colitis

The LPS challenge experiments demonstrated that both i.p. and orally-administered LMP-420 significantly inhibited *in vivo *TNF production in mice both systemically, as indicated by serum TNF and locally in the colon. Therapy with the anti-TNF antibody drug infliximab has been shown to provide clinical benefit for at least a subset in humans with IBD. To determine the efficacy of LMP-420 therapy in a murine model of IBD, C57BL/6 mice were given 3% DSS in drinking water for 7 days. Therapy with 1 mg LMP-420 or vehicle given i.p. once daily was begun on day 4 of DSS exposure, when symptoms of weight loss, decreased stool consistency, and stool bleeding indicated the onset of severe acute colitis. The 1 mg i.p. dose was chosen to minimize the hypotension and behavioral depression that was seen when 2 mg was administered in the LPS challenge study. On day 7 of DSS exposure, DSS-exposed mice treated with vehicle had markedly increased levels of colonic TNF (409 ± 107 pg/100 mg colon tissue; n = 5) compared to mice who were not exposed to DSS (65 ± 5 pg/100 mg colon tissue; n = 5) (p = 0.03). DSS-exposed mice that were treated with i.p. LMP-420 demonstrated a significant (85%) decrease in colonic TNF levels (117 ± 8 pg/100 mg tissue; n = 5; p = 0.05 vs. vehicle-treated mice), that represented near normalization of their colonic levels of TNF compared with control mice that were not exposed to DSS. TNF was not detected in the serum of either vehicle- or LMP-420-treated DSS-exposed or control mice at this time point.

### LMP-420 has no effect on histologic severity of acute or established chronic DSS colitis

**Figure 4:**
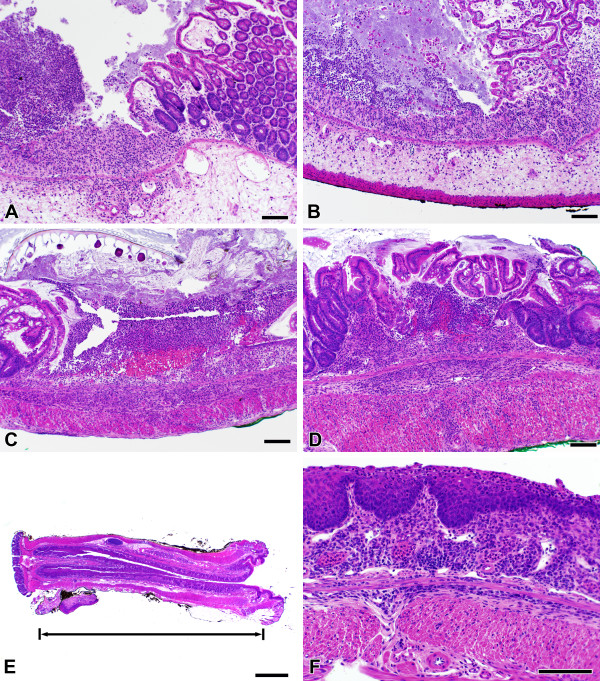
**Histologic changes during acute and chronic DSS colitis**. Colon tissues from both control (A) and LMP-420-treated (B, 15 mg/kg LMP-420 i.p.) mice with acute DSS colitis demonstrated similar amounts of edema, acute inflammatory infiltrates, and focal ulceration. Chronic colitis generated by 3 cycles of 5 days of 3% DSS in drinking water, followed by 16 days of plain water resulted in development of severe chronic colitis (C) that was not altered by a 16 day treatment with treatment LMP-420 (D, 45 mg/kg i.p.). The cecum is shown in panels A and B and mid-colon is shown in panels C and D. Wild-type and RAG-2^-/- ^mice with chronic DSS colitis developed extensive squamous metaplasia of the rectum (E, F) that in some cases extended proximally for > 1 cm. The bar equals 100 μm except for panel E, where bar = 1 mm.

Despite the efficacy of i.p. LMP-420 in decreasing colonic levels of TNF induced by DSS exposure, there was no difference in clinical or histologic severity of colitis observed on day 7 in mice treated i.p. with vehicle- vs. LMP-420 (data not shown). However, the 3 day treatment period used was likely too short to allow healing. Longer treatments were not possible in the acute DSS model due to severe weight loss that required euthanasia for humane reasons. To determine if LMP-420 treatment could prevent the development of acute DSS colitis, LMP-420 was administered i.p. for 5 days prior to as well as throughout the 7 day exposure to DSS. Oral administration of LMP-420 was not used for these studies, since mice rapidly decrease their food consumption when acute colitis develops and it was not possible to maintain a consistent oral dose using drug mixed with food. Systemic (i.p.) treatment with LMP-420 given prior to and during DSS exposure did not alter the clinical or histologic severity of colitis (Figure 4A, B). The severe acute inflammation induced by DSS tends to produce fibrosis that can be objectively monitored by measuring colon lengths. LMP-420 treatment also did not affect colonic shortening induced by DSS (Table 1).

**Table 1:** Colonic shortening induced by DSS treatment

Treatment	Colon length, cm*
No DSS	8.4 ± 0.2
DSS alone	6.5 ± 0.1
DSS/0.5 mg LMP-420 i.p.	6.6 ± 0.1
DSS/1 mg LMP-420 i.p.	6.4 ± 0.2
DSS/2 mg LMP-420 i.p.	6.6 ± 0.1

* Mean ± SEM (n = 5/group)

**Figure 5:**
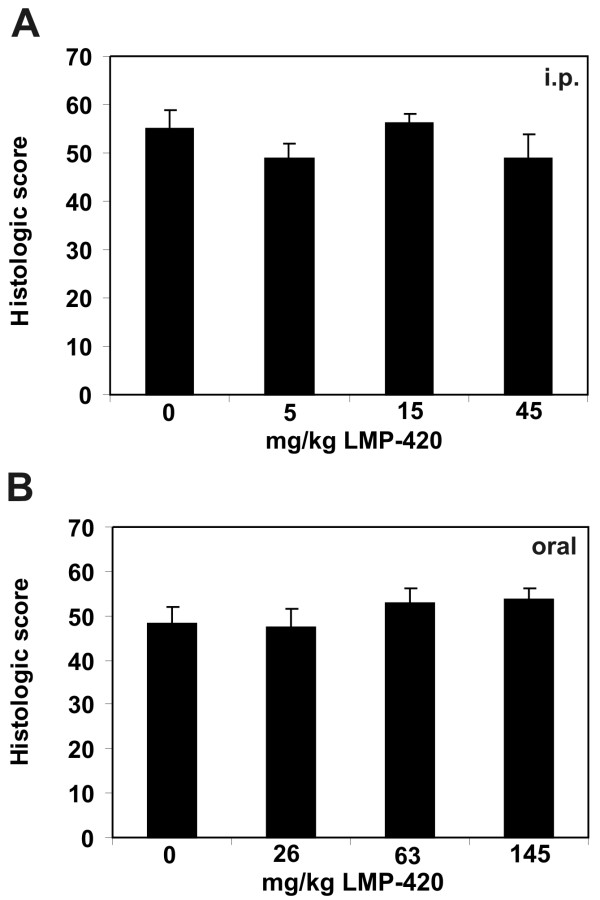
**Effect of LMP-420 in chronic DSS colitis**. Chronic colitis was generated in wild type C57BL/6 mice by 3 cycles of 5 days of 3% DSS in drinking water, followed by 16 days of plain water. LMP-420 therapy was given parenterally (i.p.) or orally in food to groups of 5 mice, beginning after completion of the 3d administration of DSS and continuing throughout the final 16 day recovery period. All mice had severe colitis at the termination of the study. Histologic scores were calculated as described. No significant differences in histologic scores were observed in mice treated with LMP-420 doses of 0 – 45 mg/kg given i.p. (panel A) or 0 – 145 mg/kg given orally (panel B).

Next, we determined whether LMP-420 treatment would influence the severity of chronic colitis initiated by multiple cycles of DSS exposure. Treatment with a dose range of LMP-420 was begun immediately upon discontinuation of the 3d DSS cycle when all mice had severe chronic colitis. The LMP-420 dose range was 0, 5, 15, 45 mg/kg/day for i.p. administration and 0, 26, 63, and 145 mg/kg/day for oral administration. Mice were euthanized after 16 days of treatment to determine colonic TNF content and the histologic severity of colitis. Although gross inflammation (edema, redness) was subjectively decreased in LMP-420-treated mice, there was no difference in histologic scores between treated and untreated mice (Figure 4C, D; Figure 5). In humans, histologic mucosal healing typically lags behind gross improvement and it is possible that longer treatment periods may yield differences in histologic scores. Very interestingly, marked squamous metaplasia of the rectum, sometime extending proximally for > 1 cm, was consistently observed in all mice exposed to 3 cycles of DSS, regardless of treatment group (Figure 4E, F). Despite the very severe inflammation, TNF levels in colonic tissue were low in all groups, including untreated control groups, and did not differ according to treatment group. The colonic TNF content of these mice was statistically similar to that present in non-DSS-exposed mice without colitis (data not shown).

### Stool TNF levels are not elevated in chronic DSS colitis

**Figure 6:**
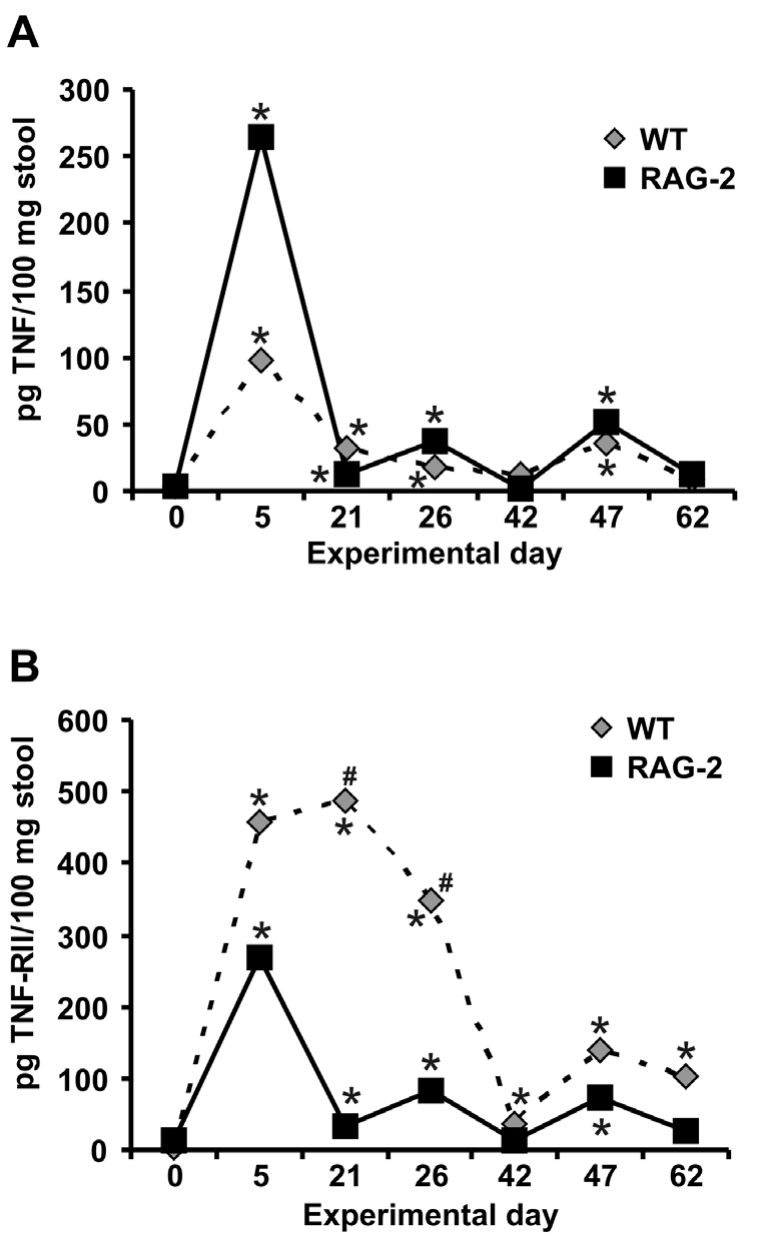
**Stool levels of TNF and TNF-RII during induction of chronic DSS colitis**. Levels of TNF (panel A) and TNF-RII (panel B) were determined by enzyme immunoassay in stool samples obtained before DSS exposure began, at the end of each 5 day cycle of DSS administration, and after 16 days of recovery prior to beginning the next DSS cycle for wild type (n = 49–50 mice for days 0, 47, and 62, and n = 9 – 10 for the remaining time points) and RAG-2^-/- ^mice (n = 14). Mean cytokine concentrations per 100 mg stool are shown. Error bars are omitted for clarity. * indicates values significantly different from pre-treatment level for a given genotype. # indicates values significantly different (p ≤ 0.02) in wild type vs. RAG-2^-/- ^mice.

The effects of LMP-420 on TNF production was profound in acute DSS colitis, but LMP-420 therapy apparently had no effect on inflammation severity in either acute or established chronic DSS colitis. Furthermore, colonic TNF levels were not elevated in untreated control mice with chronic DSS colitis, despite the presence of very severe colonic inflammation (histologic scores of 52 ± 3; n = 10). To begin to understand these observations, a longitudinal study of the levels of TNF in the stool was performed. Stool samples were obtained prior to the initial DSS exposure (day 0), at the end of each 5 day cycle of DSS administration (days 5, 26, and 47), and after 16 days of healing prior to beginning the next cycle of DSS administration (days 21, 42, and 63). Because levels of soluble TNF receptors increase during inflammation due to receptor shedding after binding TNF and/or increased alternative splicing that generates the soluble form, the levels of TNF-RII (p75, CD120b; encoded by the TNFRSF1B gene) in stool were also measured. In wild type C57BL/6 mice, TNF levels in stool were significantly increased compared to baseline levels at the end of each cycle of DSS administration. Stool TNF then spontaneously decreased to normal levels by the end of each 16 day period of recovery (Figure 6A). Stool levels of TNF-RII also increased very markedly during DSS administration and then decreased during the recovery period. However, in contrast to stool TNF, stool TNF-RII levels did not return to baseline but remained significantly elevated throughout all recovery periods (Figure 6B).

### Role of T and B lymphocytes in chronic DSS colitis

Acute DSS colitis has been shown to occur in the absence of T and B lymphocytes [28], however the establishment of chronic DSS colitis has been hypothesized to involve adaptive as well as innate immune cells. To test this, RAG-2^-/- ^mice that lack both T and B cells were exposed to the same regimen of 3 cycles of DSS that induced severe chronic colitis in wild type mice, then euthanized 16 days following the 3rd administration of DSS. All RAG-2^-/- ^mice had severe colitis after exposure to 3 cycles of DSS, with histologic scores of 53 ± 1 (mean ± SEM; n = 14). These scores were statistically similar to those observed in wild type mice exposed to a similar regimen of 3 DSS cycles, however the histologic picture was very different. In contrast to the marked mucosal hyperplasia seen in wild type mice, RAG-2^-/- ^mice exhibited minimal mucosal hyperplasia. Mucosal histologic sub-scores were high nonetheless, due to marked squamous metaplasia in the rectum and architectural distortion throughout the colon, manifested primarily by crypt branching and crypt dropout. Inflammatory infiltrates in the RAG-2^-/- ^mice consisted of a small number of mononuclear cells and a moderate to large number of neutrophils, and these infiltrates were more uniformly spread through the tissues. Frank ulcerations and crypt abcesses were present, but less common compared with the wild type mice.

RAG-2^-/- ^mice initially developed higher mean levels of TNF in their stool following DSS exposure, compared to wild type mice (Figure 6A). However, the overall pattern of TNF elevation observed after DSS administration followed by spontaneous recovery to baseline was similar to that observed in wild type mice. In contrast to the marked and consistently high levels of TNF-RII seen in wild type mice, TNF-RII levels in RAG-2^-/- ^mice very closely followed the levels of TNF, rising with DSS administration and falling to or near baseline during recovery (Figure 6B). Stool levels of TNF in RAG-2^-/- ^mice with severe chronic DSS colitis were 13 ± 6 pg/100 mg stool at the time of tissue collection on day 62, which is statistically similar to pre-DSS levels of 3 ± 1 pg/100 mg stool (p = 0.13). The TNF levels in colon tissue measured in a subset of these mice (n = 5) was 72 ± 3 pg/100 mg tissue, which is similar to that seen in control wild type mice without colitis (Figure 3A). TNF was not detectable in the serum of these mice at the time of tissue collection. Taken together, these data demonstrate that severe colitis can occur in the absence of T and B cells and without systemic or local colonic or stool elevations in TNF.

### LMP-420 in the IL-10^-/- ^model of chronic colitis

**Figure 7:**
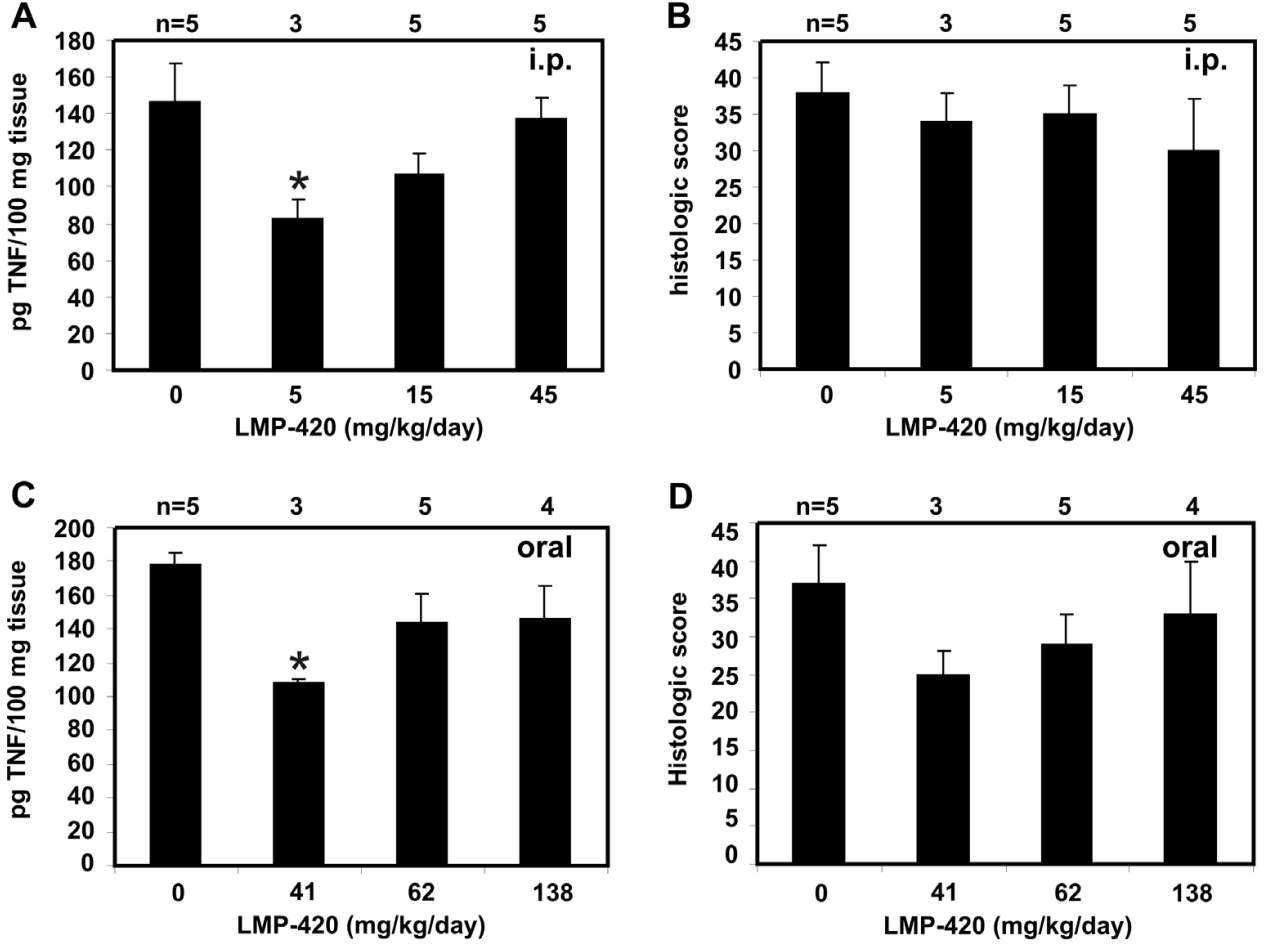
**Colonic TNF levels and histologic scores in control and LMP-420-treated IL-10^-/- ^mice with chronic colitis**. **A, C**. Levels of TNF in colonic tissues of IL-10^-/- ^mice with chronic colitis following 16 days of treatment with i.p. (panel A) or oral (panel C) LMP-420 at the indicated doses. LMP-420 doses of 5 mg/kg/day i.p. and 41 mg/kg/day oral significantly decreased total colon tissue TNF content by 44% (p = 0.03) and 39% (p = 0.0003), respectively. **B, D**. Histologic scoring of colon tissues from the same mice showed a trend toward decreased histologic evidence of inflammation in mice treated with 41 mg LMP-420/kg/day orally (panel D), however this change approached (p = 0.06) but did not reach statistical significance. No decreases in histologic scores were seen in mice treated with i.p. LMP-420 (panel B), despite the decreased colonic TNF levels observed in panel A.

Colitis has been reported to develop spontaneously in IL-10-deficient mice that are not kept germ-free, but the age of onset can vary widely between animal facilities. We used a brief exposure to the non-steroidal anti-inflammatory drug piroxicam to uniformly trigger the development of chronic colitis in 6 – 7 wk IL-10^-/- ^mice. Once colitis was established, mice were treated with a dose range of i.p. (0, 5, 15, or 45 mg/kg/day) or oral LMP-420 (0, 41, 62, or 138 mg/kg/day) for 16 days. Effects on colonic TNF and histologic severity of colitis were determined. Colonic levels of TNF were elevated in untreated IL-10^-/- ^mice with colitis compared with wild type mice without colitis (Figure 7A, C). Low doses of either i.p. (5 mg/kg/day) or oral (41 mg/kg/day) LMP-420 significantly reduced colonic tissue TNF levels by 44 or 39% respectively to near normal levels (Figure 7A, C). This TNF-lowering effect was lost at higher i.p or oral doses. Although the colons were subjectively less inflamed grossly in mice treated with i.p. LMP-420, no differences in histologic scores were seen for any of the i.p. treatment groups (Figure 7B). However, a trend toward decreased histologic score (p = 0.06) was observed in mice treated with 41 mg/kg oral LMP-420 (Figure 7D), consistent with the decreased colonic TNF observed in this group.

## Discussion

The novel small molecule drug LMP-420 is highly effective in inhibiting TNF production both *in vitro *and *in vivo*. When given to mice either i.p. or orally, LMP-420 significantly decreased both serum (-42%) and colonic TNF responses (-67%) to challenge with a lethal dose of LPS. LMP-420 treatment also reduced (-85%) TNF elevations associated with acute DSS colitis to near baseline levels. However, despite its efficacy in reducing TNF levels *in vivo*, LMP-420 had no effect on the histologic severity of colonic inflammation in response to either acute or chronic DSS administration. A trend toward decreased inflammation (p = 0.06) was observed in IL-10^-/- ^mice treated orally with LMP-420, which correlated with decreased colonic TNF levels. Histologic healing of colonic inflammatory lesions is known to lag behind clinical remission and endoscopic healing. Thus, it is possible that LMP-420 treatments of longer durations might result in beneficial clinical effects that were not observed in this pilot study using short treatment periods.

The availability of LMP-420, a small-molecule, orally-active inhibitor of TNF production, provided us an attractive opportunity to define the potential role of TNF in the murine model of DSS-induced colitis, a commonly-used model of inflammatory bowel disease. Although we were able to demonstrate significant inhibition of colon TNF by LMP-420 in this model, we had no effect on the pathological inflammation. However, in contrast to the biological TNF antagonists currently used in humans which are capable of "neutralizing" essentially all circulating TNF, LMP-420 allowed ~20% of colon TNF to be produced in our model. Thus, while our data might be interpreted to suggest that TNF does not play a significant role in this model, we cannot at this time rule out the possibility that the small amount of TNF produced was sufficient to induce pathogenesis. Alternatively, prevention of TNF synthesis without toxicity to TNF-producing cells may not be sufficient to stop the inflammatory cascade *in vivo*. Spohn *et al*. recently showed that anti-TNF antibodies that bound to both membrane-bound and soluble forms of TNF had a greater anti-inflammatory effect in a murine model of rheumatoid arthritis than antibodies reactive only with soluble TNF [29]. Furthermore, significant immunosuppression leading to reactivation of latent tuberculosis was observed only in mice with antibodies reactive with membrane-bound TNF [29]. Thus, it is possible that additional beneficial effects of TNF antagonists result from binding to membrane-bound TNF followed by cytolysis of TNF-producing cells, an activity that is present with infliximab and other antibodies that bind membrane-bound TNF, but not with etanercept or LMP-420. Given the lack of efficacy of etanercept in treating CD [17] despite its demonstrated efficacy in rheumatoid arthritis and psoriasis and the inability of LMP-420 to ameliorate murine DSS-colitis despite lowering colon TNF levels, the role of direct TNF inhibition in the pathogenesis and/or treatment of inflammatory bowel disease remains undetermined.

Levels of TNF excreted in the stool correlated well with levels of TNF measured in colon tissues harvested at the time of euthanasia, providing a method to follow colonic TNF levels non-invasively. Longitudinal measurements of TNF in the stool of mice subjected to multiple cycles of DSS demonstrated that, although TNF levels are elevated in acute DSS colitis, these levels decrease spontaneously and eventually return to baseline despite ongoing severe inflammation. Taken together with the lack of efficacy of LMP-420 in acute or chronic DSS colitis despite its ability to significantly lower colonic TNF levels, these data suggest that elevated levels of TNF are not required for the initiation and maintenance of colonic inflammation in this murine model. Our data thus is similar to that of Olson *et al*, who previously reported that TNF was not detectable in colon tissue or plasma of CBA/J mice with acute DSS colitis, and that a polyclonal anti-TNF antiserum had no effect on disease severity [30]. However, Naito *et al *showed that TNF-deficient mice have increased intestinal inflammation in response to DSS compared with wild type mice [31]. These contrasting data suggest that increased investigation will be necessary to clarify the role of TNF in DSS-induced colitis.

Our studies showed that levels of TNF and TNF-RII in the stool are highly correlated for RAG-2^-/- ^mice, in contrast to the markedly higher TNF-RII levels observed in stool of wild type mice subjected to multiple cycles of DSS. The manufacturer reports that the presence of TNF-RII does not affect the ability of its TNF ELISA assay to quantitiate TNF. Thus additional mechanisms beyond simple shedding of receptor that has bound TNF likely account for the marked and sustained increase in stool TNF-RII during chronic DSS colitis in wild type mice. The induction of severe chronic colitis following repeated DSS administration to RAG-2^-/- ^mice that lack T and B cells suggests that innate immunity is sufficient to drive the development of chronic colitis in the DSS model. Use of other models that are TNF-dependent or driven by induced T cell responses will be necessary to determine if LMP-420 may have efficacy in maintaining remission of chronic colitis driven by TNF or T cells.

The induction of extensive squamous metaplasia in the terminal colon and rectum by multiple cycles of DSS administration is an interesting pathologic observation that is of uncertain clinical significance. Metaplasia has been observed in a range of organs and is thought to occur in response to chronic irritation [32]. T and B cells are apparently not required for this histopathologic change, since similar degrees of squamous metaplasia were observed in both wild type and RAG-2^-/- ^mice. The presence of squamous mucosa is typically limited to the anus in both humans and mice. The simple columnar epithelium that is normally present in the terminal colon and rectum functions to absorb fluid from stool as well as to secrete mucus to lubricate its passage. These functions would be missing from metaplastic squamous epithelium. By analogy with other organs, it is possible that mice with extensive squamous metaplasia of the colon might be at increased long-term risk for development of squamous carcinomas of the colon rather than the adenocarcinomas that typically develop at this site. Longer term studies will be needed to address this possibility.

The effect of LMP-420 was additionally studied in the IL-10^-/- ^model of murine colitis. As we saw for acute DSS colitis, LMP-420 treatment (5 mg/kg i.p. and 41 mg/kg oral) significantly decreased colonic TNF levels in a setting where TNF was elevated in the colons of mice that did not receive this drug. However, again we saw no statistically significant decreases in severity of colitis as measured histologically, although there was a trend to decreased histologic inflammation for mice that received 41 mg/kg oral LMP-420. Increased potential effect of oral as compared with systemically administered drug suggests that LMP-420 may exhibit local anti-inflammatory activity as it passes through the gastrointestinal tract. Lack of TNF lowering in the IL-10^-/- ^model at higher LMP-420 doses indicates a complex dose-response profile that may reflect dual activity of LMP-420 in competing inflammatory/anti-inflammatory pathways.

Taken together, these studies demonstrate that short-term treatment with a transcriptional inhibitor of TNF production does not decrease the severity of acute and chronic DSS colitis or piroxicam-accelerated colitis in IL-10^-/- ^mice. A detailed dose-response study and longer treatment durations using other colitis models that are more dependent on TNF elevation should be performed to more accurately assess the potential of LMP-420 for therapy of inflammatory bowel disease.

## Abbreviations

BSA: bovine serum albumin; CD: Crohn's disease; DSS: dextran sulfate sodium; FBS: fetal bovine serum; i.p.: intraperitoneal; IBD: inflammatory bowel disease; IFN: interferon; IL: interleukin; LPS: lipopolysaccharide; MCP: monocyte chemoattractant protein; PBS: phosphate buffered saline; SD: standard deviation; SEM: standard error of the mean; TNF: tumor necrosis factor; TNF-RII: tumor necrosis factor receptor, type II; UC: ulcerative colitis.

## Competing interests

LPH has no competing interests. GJC also has no competing interests, but discloses that he was a co-discoverer of LMP-420 and was associated with LeukoMed Inc (the company that holds the license for LMP-420) until October 2005.

## Authors' contributions

LPH conceived of and designed the studies, obtained funding, performed the pathologic and data analyses, prepared figures, and drafted the manuscript. GJC assisted in study design, reviewed data, prepared figures, and helped to draft the manuscript. All authors read and approved the final manuscript.
